# Impact of the COVID-19 pandemic in the treatment of patients with acromegaly in a tertiary center: a wake-up call on the importance of telemedicine

**DOI:** 10.20945/2359-3997000000577

**Published:** 2022-11-28

DOI: 10.20945/2359-3997000000491

Arch Endocrinol Metab. 2022;66(6):863-7

Where you read:

Matheus Zaian Rodrigues de Fonseca Lira^4^

https://orcid.org/0000-0002-1270-0026

Should read:

Matheus Zaian Rodrigues de Fonseca Lira^4^

https://orcid.org/0000-0002-8720-0026

